# Activities of the Animal Welfare Body at the Istituto Zooprofilattico Sperimentale Umbria and Marche “Togo Rosati” from 2016 to 2025

**DOI:** 10.3390/ani16192977

**Published:** 2026-09-22

**Authors:** Martina Pellegrini, Giulia Vita, Roberto Sabato, Cecilia Righi, Eleonora Scoccia, Giovanni Ricci, Alessandra Martucciello, Mohamed Naceur Haouet, Elisabetta Manuali, Francesco Barretta, Anna Fratto, Giulio Severi, Stefano Petrini

**Affiliations:** 1 Istituto Zooprofilattico Sperimentale dell’Umbria e delle Marche “Togo Rosati”, Via Gaetano Salvemini 1., 06126 Perugia, Italy; g.vita@izsum.it (G.V.); r.sabato@izsum.it (R.S.); c.righi@izsum.it (C.R.); e.scoccia@izsum.it (E.S.); mn.haouet@izsum.it (M.N.H.); e.manuali@izsum.it (E.M.); f.barretta@izsum.it (F.B.); a.fratto@izsum.it (A.F.); g.severi@izsum.it (G.S.); s.petrini@izsum.it (S.P.); 2 Dipartimento di Medicina Veterinaria, Università degli Studi di Perugia, Via San Costanzo 4, 06126 Perugia, Italy; giovanni.ricci@unipg.it; 3 Istituto Zooprofilattico Sperimentale del Mezzogiorno, Via della Salute 19, 80055 Portici, Italy; alessandra.martucciello@izsmportici.it

**Keywords:** animal ethics, animal experimentation, animal welfare, animal welfare body, governance, institutional oversight, 3Rs

## Abstract

Animal welfare and ethical responsibility are central considerations in animal research. In Italy, European Directive 2010/63/EU was implemented through Legislative Decree No. 26/2014, establishing Animal Welfare Bodies (AWBs) to ensure ethical project evaluation and promote the Replacement, Reduction, and Refinement (3Rs) principles. Despite their regulatory importance, long-term analyses of AWB activities remain limited, particularly within the Italian context. This study presents a ten-year retrospective analysis of the AWB of the Istituto Zooprofilattico Sperimentale dell’Umbria e delle Marche “Togo Rosati” (IZSUM) from 2016 to 2025. The study collected data on project evaluations, species involved, opinions issued, retrospective assessments, personnel competence management, and welfare monitoring procedures. Over the study period, the AWB evaluated 33 projects, issuing 26 positive opinions, 6 positive opinions with reservations, and 1 negative opinion, and conducted 11 retrospective assessments. The data showed marked fluctuations in authorised animal numbers, including a substantial reduction in mouse authorisations between successive five-year regulatory testing cycles. These trends occurred alongside the implementation of alternative methods and changes in regulatory testing strategies. The findings describe the activities and longitudinal outcomes of a single institutional AWB and are consistent with a potential contribution to 3Rs implementation, while the single-institution observational design does not allow causal attribution of these changes to AWB activities alone.

## Simple Summary

Animal use in scientific research requires a robust ethical and regulatory framework to ensure high animal welfare standards. In Italy, this framework was implemented through transposing Directive 2010/63/EU into national legislation and establishing animal welfare bodies (AWBs). In this study, we examined the activities conducted by the AWB at the Istituto Zooprofilattico Sperimentale dell’Umbria e delle Marche “Togo Rosati” (IZSUM) over a 10-year period (2016–2025), with particular attention to project evaluation; Replacement, Reduction, and Refinement (3Rs) principles; personnel training; and animal welfare monitoring. The results provide a description of AWB activities, including project evaluations, authorised animal numbers, severity classifications, and retrospective assessments. The observed reduction in authorised animal numbers, particularly for mice between successive regulatory testing cycles, coincided with the implementation of alternative methods and changes in testing strategies. These findings are consistent with a potential contribution of institutional AWB activities to the implementation of the 3Rs, but do not by themselves establish a causal effect of the AWB.

## 1. Introduction

The use of animals in scientific research remains fundamental to veterinary and biomedical fields; however, it simultaneously raises considerable ethical and societal concerns [1,2]. To address these challenges, the 3Rs principles (Replacement, Reduction, and Refinement) provide the internationally recognised framework for minimising animal use and suffering while maintaining scientific validity [2]. Recent scientific literature has highlighted both the ethical constraints and sometimes inconsistent predictive value of traditional animal models, alongside the need for the systematic implementation of alternative and refined methodologies.

At the European level, Directive 2010/63/EU established a harmonised legal framework designed to ensure high-quality scientific research while safeguarding animal welfare [1]. The Directive sets out detailed requirements regarding housing and care standards, personnel competence, project authorisation procedures, and internal oversight body establishment. These elements are essential for cultivating a robust culture of care that transcends basic regulatory compliance, thereby fostering a shared commitment to the 3Rs principles, animal welfare, and scientific excellence [1]. In Italy, the Directive was transposed into national legislation through Legislative Decree No. 26 of 4 March 2014, which formally introduced Animal Welfare Bodies (AWBs) within institutions engaged in animal research [3]. Unlike the general framework of Article 26 of Directive 2010/63/EU, the Italian transposition (Articles 18 and 31 of Legislative Decree No. 26/2014) specifically mandates the institutional AWB to perform an obligatory preliminary evaluation of all research project proposals, issuing a formal opinion prior to their submission to the Ministry of Health for final project authorisation. The Ministry of Health Decree of 5 August 2021 and the Director-General Decree of the Ministry of Health of 18 March 2022 established subsequent national provisions on training personnel involved in animal experimentation [4,5].

AWBs play a key role in bridging scientific research and ethical oversight [6]. At the IZSUM, the AWB acts as a technical and scientific interface between researchers and the Ministry of Health, ensuring compliance with applicable regulations and adherence to animal welfare principles in research activities. The main tasks of AWB include the following: 1. Evaluating projects; 2. Monitoring of approved studies; 3. Promoting the 3Rs principles; 4. Assessing personnel training and competence; 5. Issuing reasoned opinions on research proposals; 6. Maintaining documentation related to the project evaluation and staff training and managing the animal experimentation database.

Within this framework, the IZSUM has progressively strengthened its commitment to animal welfare by implementing targeted training initiatives, fostering methodological innovation, and actively engaging in national and international networks.

The IZSUM AWB was formally established in May 2014 (Director General Resolution No. 172 of 9 May 2014) and initially comprised four members: a coordinator, an animal welfare officer, a designated veterinarian and a scientific member. Over time, the group structure has evolved substantially, leading to its current composition (Director General Resolution No. 77 of 29 February 2024). This includes, in addition to the aforementioned roles, six additional scientific members with diverse professional backgrounds—veterinarians, biologists, laboratory technicians, histopathologists, virologists, and data analysis specialists—along with staff in training. Furthermore, since 2024, a veterinarian from the experimental animal facility at the University of Perugia and a laboratory veterinarian from the IZSM of Southern Italy have joined the AWB. This expansion has strengthened the multidisciplinary nature of the group, enabling more effective management of animal welfare and more comprehensive evaluation of research proposals. Members are appointed by the Director General for a five-year term and must fulfil AWB duties, attend scheduled meetings, and participate in specific professional training and continuing education activities.

In this study, we analyse the activities of the IZSUM’s AWB over a ten-year period (2016–2025), providing an overview of its procedures, structure, and outcomes. To the best of our knowledge, this is the first ten-year retrospective analysis of an Italian AWB in veterinary public health, a sector in which long-term data remain scarce. By presenting both quantitative and qualitative findings, we aim to contribute to the international debate on the effectiveness of ethical governance and to offer useful insights for strengthening oversight systems within research organisations.

## 2. Materials and Methods

### 2.1. Institutional and Regulatory Framework

The activities described in this study were conducted in accordance with Directive 2010/63/EU and Italian Legislative Decree No. 26/2014. The IZSUM AWB operates in coordination with national authorities, including the National Committee for the Protection of Animals Used for Scientific Purposes (CNPA). This committee was established in accordance with Italian Legislative Decree No. 26/2014. Moreover, the AWB collaborates with the Italian Committee of Animal Welfare Bodies (CIO), research centres, and the Italian Ministry of Health.

The Animal Experimentation Database, managed by the Italian Ministry of Health and accessible through a dedicated online platform (stabulari.izs.it), represents a national centralised system designed to ensure transparency, traceability, and oversight of animal use in scientific research. The system centralises statistical data, authorisations, and reporting activities to facilitate monitoring of the implementation of the 3Rs principles.

To establish a standardised institutional governance framework, the IZSUM Animal Welfare Body (AWB) operates under structured Standard Operating Procedures (SOPs) and a multidisciplinary committee structure, in line with Italian Legislative Decree No. 26/2014.

The multidisciplinary AWB team at IZSUM comprises the Scientific Coordinator (who serves as AWB Chair), the Designated Veterinarian (responsible for animal health, medical care, and humane endpoint definitions), and the Animal Welfare and Care Officer, who is responsible for overseeing the appropriate care and management of animals and for supervising the proper functioning of the animal facilities. This includes monitoring environmental and housing conditions, such as ventilation, temperature, humidity, lighting, sanitation and other parameters relevant to animal welfare. The AWB includes a Biostatistician (dedicated to reviewing experimental designs, power calculations, and sample size justifications), Senior Animal Care Technicians, and institutional research scientists specialising in microbiological and immunological research.

The AWB maintains a fixed operational calendar with quarterly meetings for systematic project reviews, retrospective evaluations, and facility audits. In addition, the AWB convenes ad hoc extraordinary sessions within 10 working days to address urgent project modifications or time-sensitive research amendments. The preliminary evaluation workflow—from the Principal Investigator’s (PI) initial dossier submission to the formal issuance of the AWB’s opinion—is completed within an average of 15 to 20 working days.

Project evaluations use a standardised institutional assessment grid. This tool systematically requires PIs to justify experimental rationale and statistical sample size calculations; specify implementation of the 3Rs (explicitly detailing prospective choices regarding replacement and refinement); score severity classification (mild, moderate, severe); and develop customised monitoring protocols, including pre-defined humane endpoints.

### 2.2. Extended Operational Scope: Education, Qualifications, and Outreach

In addition to its core evaluative and oversight duties—which encompass the technical-scientific evaluation of research projects, issuing formal opinions, submitting project dossiers via the national database (BDSA), and monitoring project progression through to the final retrospective assessment—the IZSUM Animal Welfare Body (AWB) plays a pivotal role in staff education, continuous training, and institutional outreach. In line with the 3Rs principles, the AWB actively promotes and organises accredited training courses focusing on laboratory animal science, animal welfare, and alternative methods. It also facilitates practical educational internships within institutional animal facilities to support emerging research personnel. Furthermore, the AWB manages and monitors researchers’ professional qualifications by maintaining updated curricula vitae, registering training certificates, and tracking Continuing Professional Education (CPE) credits acquired under the Italian Decree of 5 August 2021. This systematic oversight ensures that research staff meet regulatory competency requirements prior to undertaking experimental functions and maintain these credentials over time. Beyond internal management, senior and highly qualified AWB members extend their expertise externally by serving as specialised consultants for external research entities and actively participating in facility inspection networks for laboratory animal housing and care standards.

### 2.3. Data Collection

Data were retrospectively collected from AWB institutional records covering the 10-year period from 2016 to 2025. The following variables were analysed:

- Number of projects evaluated per year, including the type of opinion issued (positive, positive with reservations or negative);
- Species and number of authorised animals, defined exclusively as the maximum number of animals approved by the competent authority (Italian Ministry of Health) following the preliminary AWB evaluation for each multi-year research project proposal. It is important to note that these figures represent prospective project authorisations rather than retrospective annual usage reports;
- Number of retrospective assessments conducted.

Trends were analysed descriptively due to the limited sample size. All primary records, application dossiers, and evaluation forms documenting AWB activities are securely archived within the institutional official registry system and restricted-access departmental folders. Data retrieval confirmed that institutional archives were fully complete across all ten evaluated years (2016–2025). To minimise analytical bias and subjective error, a scientific member of the AWB independently extracted the data, and the remaining committee members then audited and validated it.

Project data were systematically extracted from the final, definitive project versions approved by the Italian Ministry of Health. For multi-species protocols, each authorised proposal was classified as a single project unit, strictly adhering to the unique national authorisation code assignment. This methodology ensured continuous, consistent longitudinal monitoring of project progression without fragmentation of institutional data. For annual averages and percentage distributions, the denominators correspond strictly to the 10-year observation window (2016–2025; N = 10 years) for yearly rates and to the total number of evaluated research projects (N = 33).

### 2.4. Project Evaluation Procedures

The AWB conducted a preliminary evaluation of all research projects involving animals before submission to the Ministry of Health [1,3]. The Principal Investigator had to submit a formal request for evaluation to the AWB Coordinator, accompanied by the required documentation (refer to Legislative Decree No. 26 of 4 March 2014).

The evaluation focused on several aspects, including scientific relevance, justification for animal use, application of the 3Rs principles, suitability of the experimental design (including statistical considerations), adequacy of housing and care conditions, and competence of the personnel involved. In particular, we evaluated competence by reviewing the national training completed by personnel on animal procedures, care, and euthanasia [7,8]. Competence records (“competence booklets”) were systematically maintained and regularly updated to document education, training, practical experience, and continuing professional development credits. Competence assessments were conducted in accordance with the Ministry of Health Decree of 5 August 2021 and the Ministry of Health Directorial Decree of 18 March 2022.

During the preliminary project evaluation, the AWB systematically reviews the welfare monitoring protocols submitted by the Principal Investigator (PI) within the project proposal, ensuring that species-specific clinical scoring systems, welfare indicators, and predefined humane endpoints are appropriately defined prior to project submission.

Based on this assessment, the AWB issued a reasoned opinion that could be classified as positive, positive with reservations, or negative.

When the AWB issued a conditional positive opinion, it granted project approval subject to implementing specific requested amendments or additions (e.g., protocol refinements, improved housing conditions, revised power analysis, or additional training). The AWB Coordinator communicated the opinion and required amendments to the Principal Investigator. The AWB then re-evaluated the revised project and could request further modifications or issue a final positive opinion.

A negative opinion from the AWB precluded the authorisation or execution of the protocol within the institution’s facilities. In such cases, the Principal Investigator was formally notified of the rejection, together with the ethical and scientific justifications (e.g., non-compliance with the 3Rs principles, excessive harm to animals, the use of outdated methodologies that could be replaced by alternative approaches). The Principal Investigator could revise the project by incorporating the requested amendments and resubmit it for a new evaluation.

Once the AWB completed its assessment and finalised all documentation, the project was submitted electronically to the Italian Ministry of Health via the Animal Experimentation Database. The Ministry relies on the technical and scientific assessments carried out by the Istituto Superiore di Sanità (ISS) for the scientific evaluation, whilst simultaneously conducting an administrative and documentary review. During the assessment process, both the ISS and the Ministry of Health may request further information and clarifications, which are sent to the principal investigator via the AWB coordinator. Any amendments made must be resubmitted to the AWB for assessment before being sent to the ISS and/or the Ministry of Health. Upon completing the evaluation, the Ministry of Health grants authorisation for the animal experimentation project. Depending on the severity of the procedures and duration of the project, the authorisation period may range from one to five years. The final outcome of the authorisation process, regardless of being positive or negative, is formally communicated to the Principal Investigator (Figure 1).

Projects authorised by the Ministry of Health were conducted at the IZSUM animal facilities, which included sections for housing small and large animals classified as conventional and Biosafety Level 3 (BSL-3) animal facilities, respectively.

### 2.5. Animals

Animals were routinely monitored using standardised operational procedures for clinical assessment, humane endpoints, and environmental oversight. Clinical score sheets and predefined humane endpoints were implemented in strict accordance with current Italian animal welfare regulations (Legislative Decree No. 26/2014) and national reference guidelines. Environmental parameters (temperature and relative humidity) were continuously monitored and logged in real time through a digital sensor system equipped with environmental probes. The system features automated real-time alerts sent directly to the Animal Welfare and Care Officer if any parameter deviates outside established normative ranges, allowing prompt procedural intervention [9,10].

Data were recorded in secure digital databases accessible only to authorised personnel and subject to inspection by the competent authorities.

### 2.6. Retrospective Evaluation

Retrospective evaluations were exclusively performed for projects involving procedures classified as moderate to severe. Upon completion of the experimentation, the AWB submitted the retrospective assessment.

The retrospective assessment of a research project is conducted by the Ministry of Health and relies on the documentation provided by the Principal Investigator. The assessment centres on three main aspects: achievement of the project’s initial objectives; the species and number of authorised animals, the level of harm inflicted, and the severity of the experimental procedures applied; and elements that may contribute to the further application of the 3Rs principles in animal research.

## 3. Results

### 3.1. Tracking of Staff Qualifications and Continuous Education

Over the 10-year period analysed, the AWB compiled and established official competency records for a total of 38 research and technical personnel involved in animal procedures. Currently, dedicated AWB personnel digitally manage ongoing monitoring of staff qualifications. This system streamlined the tracking process by requiring mandatory annual reports on completed educational activities and the submission of earned training certificates. Furthermore, the AWB proactively sends automated notifications to individual staff members regarding upcoming credit expirations, ensuring timely renewal of Continuing Professional Education requirements under national legislative standards and maintaining uninterrupted institutional compliance.

### 3.2. Project Evaluation

From 2016 to 2025, the AWB evaluated 33 research projects. Among these, 26 (78.78%) received a positive opinion, 6 (18.18%) received a positive opinion with reservations, and 1 (3.03%)—evaluated in 2016—received a negative opinion. Specifically, the conditional opinions were issued due to specific deficiencies identified during the review process, including: (i) incomplete or inadequate personnel training qualifications; (ii) an inappropriate number of animals; statistical analysis was often lacking to determine the ‘minimum viable number’ required to achieve the study’s objectives; and (iii) the need to adjust proposed euthanasia methods and pharmacological protocols to ensure compliance with authorised agents for the specific species involved. Furthermore, the single rejected project involved dogs; this negative opinion was grounded in the strict regulatory restrictions imposed by Italian Legislative Decree No. 26/2014 (Article 10), which generally prohibits the use of dogs, cats, and non-human primates unless exceptionally justified under highly specific legal conditions. The Ministry of Health subsequently authorised all projects with positive or conditional opinions after they implemented the requested amendments. In contrast, the single project with a negative opinion was blocked at the AWB stage and did not proceed to the Ministry of Health’s evaluation process. Table 1 indicates that most projects evaluated during the study period (2016–2025) received positive opinions, either unconditional or conditional, reflecting an iterative refinement process facilitated by the AWB (Table 2).

The annual distribution of evaluated research projects from 2016 to 2025 demonstrates notable fluctuations over the ten-year period (Figure 2). Activity peaked in 2017 with 9 approved projects, then fell sharply in the following years, remaining low between 2018 and 2020 (2 to 3 projects per year) and reaching a low of 1 project in 2021. Activity temporarily resurged in 2023 with 7 approved projects, before stabilising at lower levels in 2024 (2 projects) and 2025 (3 projects). Overall, 32 projects were approved during this timeframe, averaging approximately 3.20 approvals per year. Regarding the severity classification of the evaluated research proposals (classified in accordance with Directive 2010/63/EU into mild, moderate, and severe categories), the projects were distributed almost evenly across all three distress levels. Of the total evaluated proposals (*n* = 33), severe procedures represented the largest proportion, with 12 projects (36.36%), followed closely by mild procedures with 11 projects (33.33%) and moderate procedures with 10 projects (30.30%).

### 3.3. Species and Authorised Animal Numbers

The projects involved several animal species, including mice (*Mus musculus*), rats (*Rattus norvegicus*), guinea pigs (*Cavia porcellus*), rabbits (*Oryctolagus cuniculus*), pigs (*Sus scrofa domesticus*), wild boar (*Sus scrofa*), cattle (*Bos taurus*), hares (*Lepus europaeus*), and poultry (*Gallus gallus domesticus*).

Regarding the total number of authorised animals during the observed period, an overall total of 27,287 animals across nine species was approved between 2016 and 2025 (Table 3). Laboratory rodents accounted for most of all authorised animals, with mice representing 91.06% (n=24,850) of the overall total, followed by pigs (n=979;3.59%), guinea pigs (n=705;2.58%), chickens (n=497;1.82%), and hares (n=109;0.40%). Smaller numbers were approved for wild boars (n=42), rats (n=40), rabbits (n=35), and cattle (n=30).

The temporal breakdown of authorised animals by species demonstrates marked annual variations in both volume and species diversity (Table 4). High species diversity was concentrated in specific years, most notably in 2023, which involved seven distinct animal species (pigs, n=342; mice, n=2400; guinea pigs, n=180; wild boars, n=36; rabbits, n=15; hares, n=15; and chickens, n=10), followed by 2017 with five species (mice, n=22,440; guinea pigs, n=525; chickens, n=166; pigs, n=108; and hares, n=18). In contrast, other years exhibited a narrower focus, such as 2021 (chickens only, n=96) and 2024 (pigs only, n=248). Non-rodent species like swine and poultry showed consistent representation, with pigs being authorised in six of the nine evaluated years (peaking at n=342 in 2023) and chickens in four years (peaking at n=225 in 2019). Wild ungulates and lagomorphs appeared sporadically, with wild boars recorded only in 2023 (n=36) and 2025 (n=6), and hares peaking in 2020 (n=76).

### 3.4. Retrospective Evaluations

Retrospective evaluations (Table 5) were performed for projects that had reached completion, focusing on scientific outcomes, actual animal use, and potential applications of the 3Rs principles.

During the study period, 11 retrospective evaluations were completed, specifically for projects categorised as mild or moderate severity and concluded by the end of 2024. Although statutory mandatory retrospective assessments for NHP or severe procedures accounted for 0 cases, the competent authority and AWB requested all 11 assessments as an institutional oversight mechanism. These assessments evaluated the achievement of scientific objectives, the actual number and species of animals used, the severity of the procedures performed, and the potential for further application of the 3Rs principles. The retrospective evaluations confirmed that, in all cases, the projects achieved their primary scientific objectives, used the number of animals requested in the authorisation phase, and the animals experienced severity levels consistent with those originally predicted.

## 4. Discussion

The 10-year retrospective review of the activities conducted by the IZSUM AWB offers a descriptive overview of the operational workflow and institutional governance of animal research at the facility. The evaluation of 33 project proposals and 11 retrospective assessments reflects the integration of AWB oversight into the institution’s administrative and ethical evaluation procedures. This operational experience illustrates the role of the AWB not merely as a regulatory requirement, but as a structured mechanism supporting continuous refinement, methodological quality, and the promotion of a culture of care, in line with the objectives established for animal welfare bodies at the European level [1,6].

The AWB supports this culture through collaborative leadership, ongoing guidance on 3Rs principles, and a proactive approach that aligns animal welfare with scientific rigor [1].

A key finding is the progressive consolidation of the AWB’s evaluative function. The high proportion of positive opinions, along with a substantial number of positive opinions with reservations, reflects a process characterised by dialogue and iterative refinement rather than mere approval or rejection. Conditional approvals allow the AWB to request targeted modifications, including improved experimental design, enhanced welfare monitoring, and stronger justification of animal numbers, thereby improving project quality prior to formal authorisation. This approach aligns with international recommendations that highlight the advisory and supportive role of AWBs, rather than viewing them purely as administrative checkpoints [6,10,11].

To present the findings of this evaluation process, we analysed the protocols reviewed by the institution’s Animal Welfare Body (AWB) to characterise the nature and scope of the requested modifications. As summarised in Table 1, the iterative review of representative research projects required an average of 1.8 evaluation cycles, reflecting a structured and thorough oversight mechanism. These structured evaluations systematically addressed critical aspects of experimental design, personnel compliance, and procedural safety, ensuring every protocol aligned with high ethical and operational standards before final authorisation.

Specifically, the reservations issued by the AWB focused on two fundamental pillars of the 3Rs framework:

- Refinement and Animal Welfare Enhancements: A primary category of reservations concerned staffing and operational oversight. The AWB requested mandatory amendments to ensure that research personnel were not only fully trained and qualified but also available in sufficient numbers to guarantee round-the-clock, continuous monitoring of housed animals. These conditions directly enhanced animal welfare through improved clinical observation, early detection of distress, and strict implementation of humane endpoints.
- Reduction Measures: The remaining reservations pertained to sample size justifications. Following the AWB’s specific recommendations and feedback, researchers recalculated group sizes, resulting in revised project submissions requesting the minimum number of animals necessary to achieve valid scientific outcomes. All project proposals receiving conditional approval underwent a rigorous iterative review process, demonstrating how targeted AWB feedback actively leads to measurable improvements in welfare monitoring protocols and experimental design.

During the study period (2016–2025), the evaluated projects demonstrated a substantial reduction in the number of authorised animals, consistent with the 3Rs principles, particularly among the most frequently used species.

The temporal analysis of authorised animal numbers from 2016 to 2025 shows marked fluctuations, largely driven by the five-year authorisation cycles of specific institutional regulatory tests. The notable peaks in 2017 and 2023 (Table 4) directly correspond to the renewal of authorisations for standardised procedures, particularly those involving mice and guinea pigs. In 2017, a massive authorisation of 22,440 mice and 525 guinea pigs was recorded, while in 2023, the numbers were 2400 mice and 180 guinea pigs. These cycles are intrinsically linked to the mandatory batch testing required by the Italian Pharmacopoeia XII ed., specifically potency testing of Purified Protein Derivative (PPD) tuberculin (using guinea pigs) and residual pathogenicity testing for autogenous vaccines (using mice and guinea pigs). Because these overarching project authorisations span a five-year period, the sheer volume requested in the initial approval year artificially inflates the annual count, creating sharp peaks followed by years with negligible authorisations for these species, such as zero mice authorised from 2019 to 2022 and from 2024 to 2025.

Beyond these cyclical peaks, a critical trend over the decade is the absolute reduction in the number of animals authorised during these renewal cycles, demonstrating a strong institutional commitment to the principle of Replacement within the 3Rs framework. This reduction is most striking in mice, where the five-year cyclical request plummeted from 22,440 animals in 2017 to 2400 animals in 2023—an almost 90% decrease. Similarly, the number of guinea pigs authorised for these five-year cycles decreased from 525 in 2017 to 180 in 2023.

Despite relatively constant annual production of autogenous vaccines and associated batches from 2017 to the present—which, under traditional testing regimens, would have required a comparable number of authorised mice across both multi-year authorisation cycles—the 2023 renewal showed a sharp reduction in requested and authorised mice. This drastic reduction in mouse model use occurred alongside the successful implementation of alternative in vitro methods that have replaced traditional in vivo tests. Specifically, the decline reflects the complete replacement of the classic biological lethality test in mice with cell-based assays, such as the Endopep-MS assay or specific ELISAs [12]; furthermore, the adoption of a new, animal-free alternative method (the Limulus Amoebocyte lysate test (LAL)) for assessing residual pathogenicity in Gram-negative autogenous vaccines has substantially reduced the use of animals, replacing the in vivo residual pathogenicity test in mice [12,13]. These methodological shifts, consistent with the AWB evaluation process, illustrate how integrating alternative methods corresponds to a massive, quantifiable reduction in laboratory animal use. These methodological shifts and operational procedures highlight how the IZSUM AWB framework provides a concrete institutional example and an operational framework for animal research governance. The practical application of this operational model is reflected by key measurable outputs achieved over the 10-year period: a 100% successful completion of the institutional evaluation workflow for authorized projects, with zero post-approval protocol revocations or formal objections by national competent authorities; a 100% concordance rate in retrospective assessments (for all completed moderate and severe projects through 2024) regarding predicted versus actual severity categories and requested versus actually used animal numbers; a quantifiable ~90% reduction in mouse authorizations over successive 5-year regulatory testing cycles, driven by proactive AWB guidance towards validated in vitro alternative assays (Ph. Eur. monographs).

The systematic need for statistical justification, the promotion of alternative or complementary methods, and the refinement of procedures and housing conditions collectively contributed to minimising animal use while maintaining scientific robustness. These findings align with those of previous studies demonstrating that structured ethical review processes can effectively reduce both the number of authorised animals and the severity of procedures while maintaining research quality and outcomes [11,14].

Retrospective assessment further strengthened this governance framework. Evaluating completed projects enabled the AWB to verify whether the anticipated scientific benefits and welfare safeguards were achieved in practice, thereby closing the feedback loop between project planning and implementation. This approach aligns with recommendations highlighting retrospective assessment as a key mechanism for promoting continuous improvement, transparency, and accountability in animal research oversight [6,8]. Although retrospective assessment is inconsistently implemented across all European institutions, the experience of the IZSUM AWB demonstrates its value as a mechanism for identifying critical issues, disseminating best practices, and informing future project evaluations.

Another relevant aspect highlighted by this study is the progressive expansion of the multidisciplinary composition of the AWB. Over time, the inclusion of professionals with diverse scientific and technical backgrounds has improved the depth and breadth of project evaluations, particularly for complex studies involving multiple species or advanced methodologies. This multidisciplinary approach facilitates a more comprehensive assessment of scientific relevance, welfare implications, and the feasibility of refinement measures, thereby reinforcing the credibility and robustness of AWB decisions. This finding is consistent with that of previous research highlighting the advantages of incorporating biological, veterinary, behavioural, and methodological expertise in animal welfare assessment and ethical review [9,15].

Training and competence management also emerged as additional pillars of the governance system. The systematic verification and updating of competence records ensured compliance with regulatory requirements while maintaining high professional standards among personnel involved in animal procedures. This process not only ensured regulatory compliance but also promoted a shared culture of responsibility and awareness regarding animal welfare. Similar findings have been reported in the literature, where mandatory training and continuing professional development correlate with improved experimental practices and increased compliance with the 3Rs principles [4,5,7,8].

This study has several limitations that should be considered when interpreting the findings. First, the analysis was conducted at a single institution and therefore reflects the specific organisational, regulatory and research context of IZSUM. Consequently, the findings cannot be directly generalised to other institutions or national settings. This limitation is particularly relevant because, although Animal Welfare Bodies (AWBs) operate within the common regulatory framework established by Directive 2010/63/EU, their organisational structures, responsibilities and operational practices may vary between institutions and Member States. The international literature on AWBs remains relatively limited, with published empirical case studies describing the practical functioning of individual institutional bodies being scarce [16].

Second, although the 10-year dataset provides longitudinal quantitative information on project evaluations, authorised animals, species, severity classifications and retrospective assessments, the observational nature of the study and the predominantly descriptive indicators do not allow causal attribution of changes in animal numbers or welfare-related practices to AWB activities alone. In particular, the assessment of a “Culture of Care” and of the effectiveness of AWB-related activities remains methodologically challenging, and the literature has highlighted the need for objective, animal-centred and 3Rs-based indicators that can be measured and potentially used for benchmarking across establishments [17]. For example, the reduction in authorised mouse numbers observed between successive authorisation cycles occurred in parallel with changes in regulatory testing strategies and the implementation of alternative in vitro methods. Therefore, this finding should be interpreted as a longitudinal institutional trend that is consistent with changes in 3Rs implementation, rather than as a direct measure of AWB effectiveness. Similarly, the observed modifications in project design and welfare-related requirements provide evidence of AWB activity but should not be interpreted as proof of an independent causal effect on animal welfare or animal reduction. This cautious interpretation is consistent with the previous literature emphasising that AWBs have an important oversight and advisory role in promoting animal welfare and the practical implementation of the 3Rs, while also recognising the need for measurable indicators to assess their outcomes [14,17]. In addition, quantitative comparisons with other national or international institutions were not performed. Future multicentre and longitudinal studies using standardised indicators—such as changes in authorised and actually used animal numbers, refinement measures, implementation of replacement methods, severity outcomes, protocol amendments and retrospective assessment findings—would be valuable to determine the reproducibility and generalisability of these observations and to enable more robust benchmarking of AWB activities across institutions [17].

Overall, the findings of this study indicate that a well-structured and proactive AWB can substantially enhance the alignment between scientific advancement and high ethical and welfare standards. In this context, the IZSUM AWB experience may serve as a reference model for other research institutions seeking to strengthen their internal governance systems in line with European legislation and international best practices [1,2,6].

The evaluation workflow established by IZSUM AWB between 2016 and 2025 can provide key empirical insights into the international debate surrounding the efficiency and transparency of ethical review processes. Our institutional case study provides evidence of an adaptive, dialogue-based system in which 18.18% of projects were refined iteratively before receiving final approval, addressing the urgent need for transparent regulatory systems to address concerns regarding approval rates in animal research—a point also emphasised by Vogt and Würbel [18]. This collaborative approach is similar to that identified by Albanese et al. [19] in regional ethics committees, demonstrating that a well-structured institutional body can reconcile strict compliance with regulations and concrete scientific needs. Furthermore, while Törnqvist et al. [20] highlighted that legislative requirements and risk-benefit analyses are often regarded as rigid administrative obstacles or applied inconsistently across Europe, our decade-long dataset shows that mouse and guinea-pig authorisations were substantially lower in the 2023 renewal than in the 2017 renewal, reflecting changes in regulatory testing cycles and alternative methods.

Continued efforts toward national harmonisation, enhanced training, and methodological innovation remain essential for advancing ethical and scientific standards in animal research. Rather than capturing broad national reporting trends [21], our decade-long dataset documents the operational reality of an Italian AWB operating within the network of Istituti Zooprofilattici Sperimentali. This institutional setting shows how internal governance can adapt to a multidisciplinary research portfolio—spanning wildlife diagnostics, livestock health, and veterinary public health—while using retrospective evaluations to verify project execution, severity concordance, and ongoing 3Rs application over time.

## 5. Conclusions

From 2016 to 2025, the AWB of the IZSUM played a key role in ensuring compliance with European and national regulations, promoting the implementation of the 3Rs principles, and protecting animal welfare in scientific research. The observed substantial decrease in animal authorisations between successive regulatory testing cycles, the strengthening of multidisciplinary expertise within the AWB, and effective coordination with national authorities highlight the value of a structured institutional governance model. This approach shows how research institutions can effectively integrate ethical oversight, scientific quality, and regulatory compliance. Overall, the experience of the IZSUM AWB suggests that a proactive and multidisciplinary governance framework can substantially contribute to improving both ethical and scientific standards in animal research.

This ten-year institutional experience demonstrates that a structured, multidisciplinary AWB can effectively integrate ethical responsibility with scientific excellence. The governance model described here may serve as a useful reference for other research institutions seeking to strengthen animal welfare oversight and promote a sustainable culture of care in accordance with European standards.

## Figures and Tables

**Figure 1:**
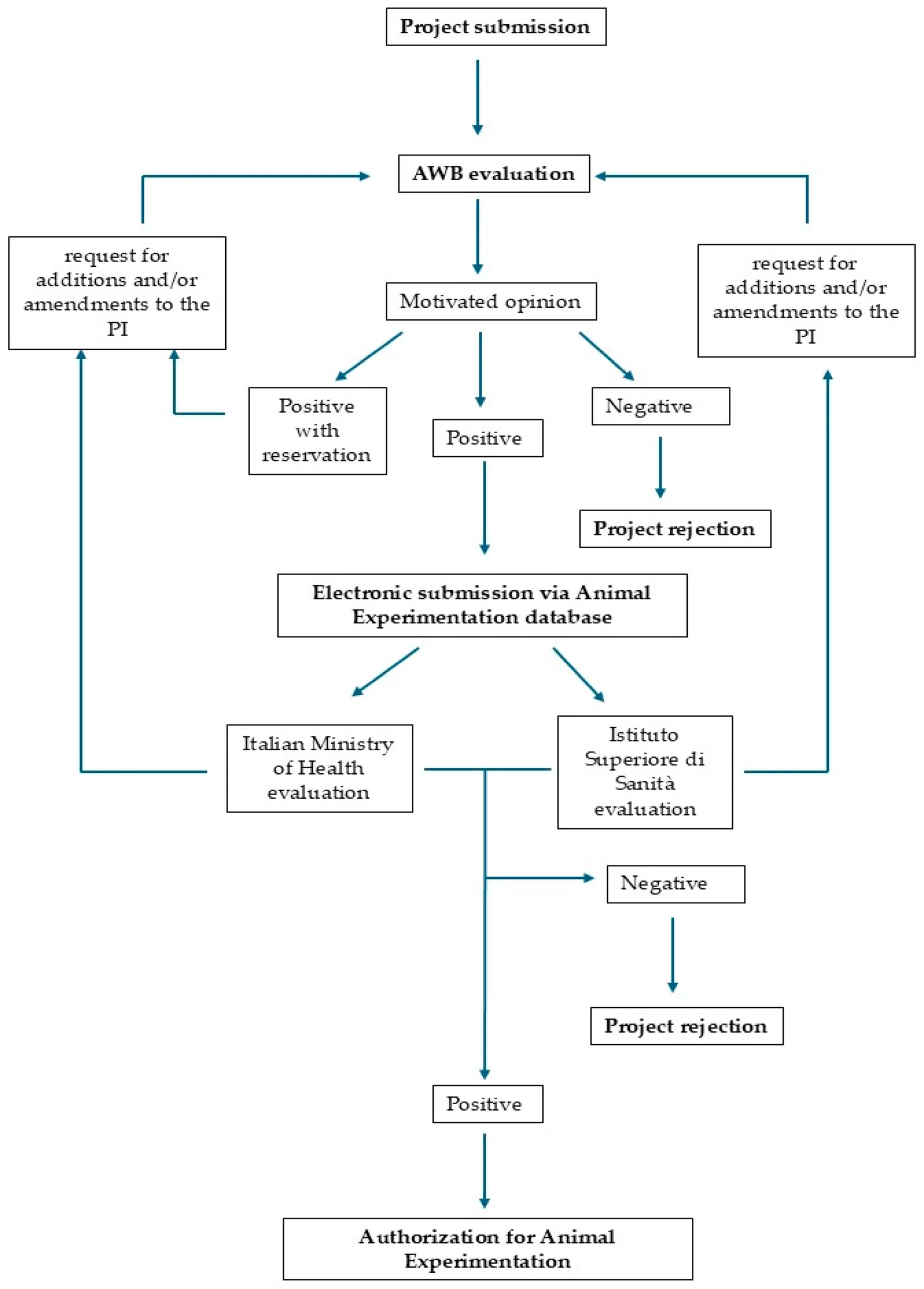
Flowchart of the evaluation and authorisation process for animal experimentation projects. AWB: Animal Welfare Body; PI: Principal Investigator.

**Figure 2:**
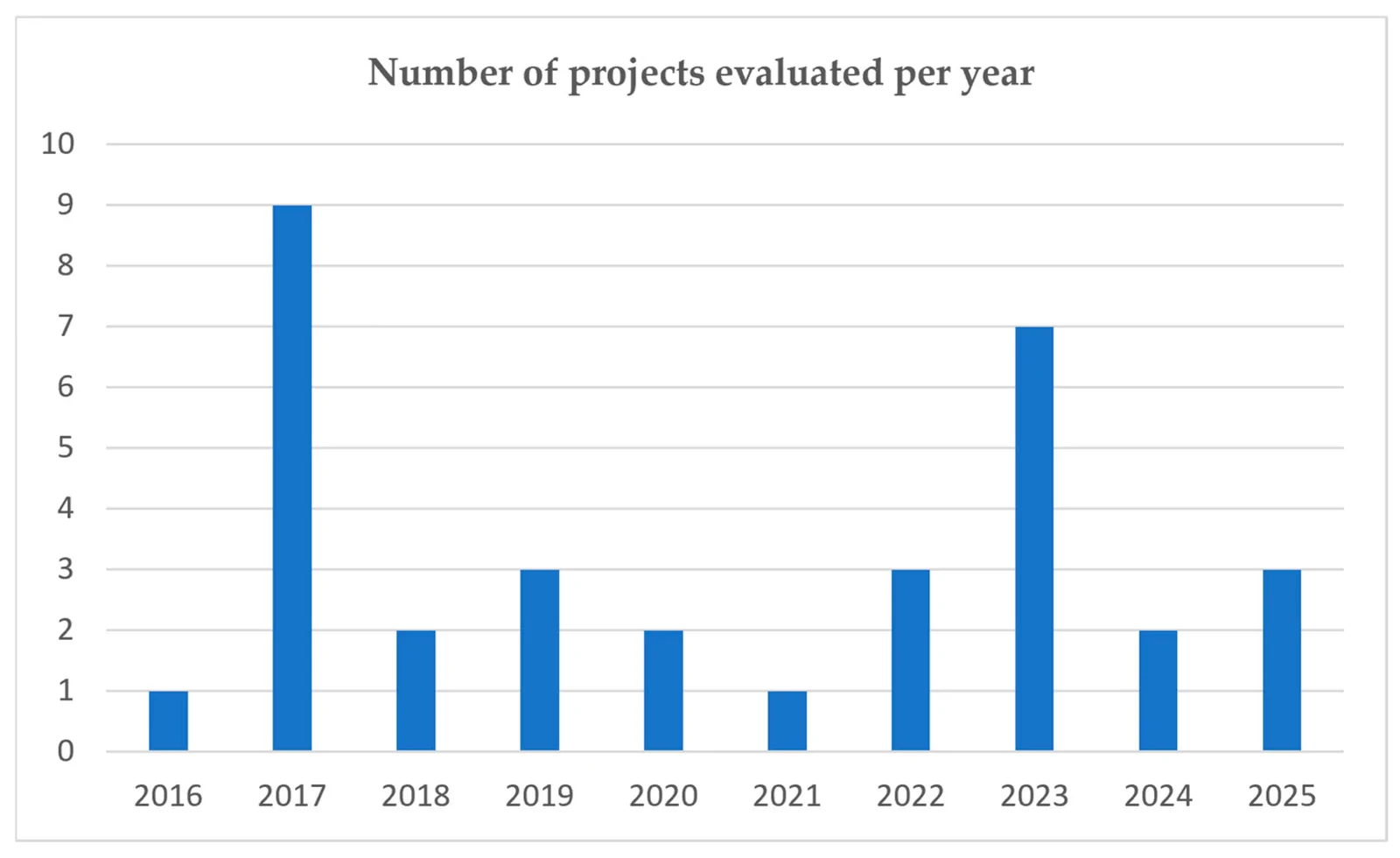
Annual distribution of approved research projects (2016–2025). The bar chart illustrates the number of research projects evaluated per year following the Animal Welfare Body (AWB) evaluation process, highlighting temporal trends and fluctuations across the ten-year study period (N=33 total projects).

**Table 1:** Summary of research projects evaluated by the IZSUM AWB (2016–2025).

Opinion Issued	Number of Projects	Percentage (%)
Positive	26	78.78
Positive with reservation	6	18.18
Negative	1	3.03
Total	33	100

**Table 2:** Overview of the representative research projects reviewed by the Animal Welfare Body (AWB), detailing the categories of requested modifications, the number of review cycles, pre- and post-review animal numbers, specific adjustments to anaesthesia, euthanasia, and personnel training, and final outcomes.

N	Categories of Modifications	Number of AWB Review Cycles	Initial vs. Final Number of Animals	Modifications Regarding Euthanasia, Anaesthesia, and Analgesia	Modifications Regarding Personnel and Training	Final Outcome
1	Title and keywords Animal house data and authorisations Drugs and culling protocols Administrative aspects and risk assessment forms	At least 2–3 review cycles and progressive integrations	Not specified	Standardise sedative drugs (Tiletamine-zolazepam and Medetomidine). Insert Atipamezole as an emergency antagonist. Clarify discrepancies regarding the culling method.	Integration of missing training certificates for the staff. Verification of the specific training pathway.	Approved
2	Experimental design and numerical reduction (3Rs) Inoculation site and heavy swine logistics Financial coverage	2 main in-depth review cycles	Reduced: Initially planned for a high number (4 per group), reduced to 3 pigs per group.	Elimination of the use of anaesthetics on animals undergoing active experimentation, favouring rapid slaughter with a captive bolt gun and exsanguination. Clarification on emergency cases.	Increase in dedicated staff (functions C and D) for BSL3 and holiday shifts. Inclusion of operators in training and planning of IZSLER practical courses.	Approved
3	Animal house authorisation details Transitional rules Technical details (electroporation, thermal cameras, and severity reclassification)	2 internal review cycles	Unchanged in conceptual design, with group remodulation	Introduction of non-invasive thermal cameras to limit stress and handling during rectal temperature measurement. Adjustment of severity classification to “Severe”.	Removal of codes unrelated to the species in the transitional rules. Correction of staff training codes.	Approved
4	Takeover and replacement of the scientific project manager Adaptation of housing temperature Review of humane endpoints (HEP), severity, and completion of the anaesthesia table	2 review and document update cycles	Unchanged in number of animals	Completion of the anaesthesia table with the introduction of the active ingredient (azaperone).	Formal replacement of the project manager. Verification of transitional rules.	Approved
5	Simplification of keywords Reconfiguration of responsibility figures Numerical animal integration, sampling schedules, human endpoint score tables, analgesic doses, and adverse reactions	1 cycle of in-depth review and request for corrections	Increased: Went from 16 to 18 (to optimise the harm/benefit balance).	Detailed inclusion of painkillers/analgesic with relative doses and administration routes. Inclusion of human endpoint (HEP) score sheets with monitoring of rectal temperatures and adverse reactions.	Rigorous adjustment of staff training	Approved
6	Keyword replacement Separation of responsibility functions and tabular competencies Insertion of hare weight, experimental design, blood volumes, and human endpoint score sheets Reclassification of severity to “Moderate” and update on reuse	1 review cycle and submission of corrective observations	Unchanged in planned numbers	Review of suffering profiles and inclusion of human endpoint score sheets and monitoring of adverse effects.	Remodulation and separation of management roles and guarantee of personnel training compliance in accordance with current legislation.	Approved

**Table 3:** Species involved in authorised research projects (2016–2025).

Common Name	Scientific Name	Number of Animals	Percentage (%)
Mouse	*Mus musculus*	24.850	91.06
Rat	*Rattus norvegicus*	40	0.15
Guinea pig	*Cavia porcellus*	705	2.58
Rabbit	*Oryctolagus cuniculus*	35	0.13
Pig	*Sus scrofa domesticus*	979	3.59
Wild boar	*Sus scrofa*	42	0.15
Cattle	*Bos taurus*	30	0.11
Hare	*Lepus europaeus*	109	0.40
Chicken	*Gallus gallus domesticus*	497	1.82
**Total**		**27.287**	**100**

**Table 4:** Annual breakdown of authorised animal numbers by species (2016–2025).

	2016	2017	2018	2019	2020	2021	2022	2023	2024	2025
Cattle	0	0	18	0	0	0	12	0	0	0
Guinea pig	0	525	0	0	0	0	0	180	0	0
Wild boar	0	0	0	0	0	0	0	36	0	6
Rabbit	0	0	2	0	0	0	18	15	0	0
Hare	0	18	0	0	76	0	0	15	0	0
Chicken	0	166	0	225	0	96	0	10	0	0
Rat	0	0	0	0	0	0	40	0	0	0
Pig	0	108	0	20	57	0	0	342	248	204
Mouse	0	22.440	10	0	0	0	0	2.400	0	0
**Total**	**0**	**23.257**	**30**	**245**	**133**	**96**	**70**	**2.998**	**248**	**210**

**Table 5:** Retrospective assessments conducted by the IZSUM AWB.

Parameter	Value
Projects subject to retrospective assessment	11
Mandatory retrospective assessments (severe procedures/NHP)	0
Voluntary or requested retrospective assessments	11

## Data Availability

The data presented in this study are derived from internal institutional archives and official registry systems of the Istituto Zooprofilattico Sperimentale dell’Umbria e delle Marche “Togo Rosati” (IZSUM). The datasets are not publicly available due to institutional privacy and administrative restrictions. However, anonymized/aggregated data are available from the corresponding author upon reasonable request and subject to institutional approval.

## Abbreviations

The following abbreviations are used in this manuscript:

3Rs	Replacement, Reduction, and Refinement
AWB	Animal Welfare Body
CIO	Italian Committee of Animal Welfare Bodies
CNPA	National Committee for the Protection of Animals Used for Scientific Purposes
ISS	Istituto Superiore di Sanità
IZSUM	Istituto Zooprofilattico Sperimentale dell’Umbria e delle Marche “Togo Rosati”
